# Generation of Rat Embryonic Germ Cells via Inhibition of TGFß and MEK Pathways

**DOI:** 10.22074/cellj.2016.3732

**Published:** 2015-07-11

**Authors:** Alireza Mohammadi, Farnoosh Attari, Vahab Babapour, Seyedeh-Naﬁseh Hassani, Najmehsadat Masoudi, Abdolhossein Shahverdi, Hossein Baharvand

**Affiliations:** 1 Department of Physiology and Pharmacology, Science and Research Branch, Islamic Azad University, Tehran, Iran; 2 Department of Stem Cells and Developmental Biology at Cell Science Research Center, Royan Institute for Stem Cell Biology and Technology, ACECR, Tehran, Iran; 3 Department of Genetics at Reproductive Biomedicine Research Center, Royan Institute for Reproductive Biomedicine, ACECR, Tehran, Iran; 4 Department of Embryology at Reproductive Biomedicine Research Center, Royan Institute for Reproductive Biomedicine, ACECR, Tehran, Iran; 5 Department of Developmental Biology, University of Science and Culture, ACECR, Tehran, Iran

**Keywords:** Pluripotency, Rat, TGFβ Pathway

## Abstract

**Objective:**

Embryonic germ (EG) cells are the results of reprogramming primordial germ cells (PGC) *in vitro*. Studying these cells can be of benefit in determining the mechanism by which specialized cells acquire pluripotency. Therefore in the current study we have tried to derive rat EG cells with inhibition of transforming growth factor-β (TGFβ) and mitogen-activated protein kinase kinase (MEK) signaling pathways.

**Materials and Methods:**

In this experimental study, rat PGCs were cultured under feeder free condition with two small molecules that inhibited the above mentioned pathways. Under this condition only two-day presence of stem cell factor (SCF) as a survival factor was applied for PGC reprogramming. Pluripotency of the resultant EG cells were further confirmed by immunofluorescent staining, directed differentiation ability to neural and cardiac cells, and their contribution to teratoma formation as well. Moreover, chromosomal stability of two different EG cells were assessed through G-banding technique.

**Results:**

Formerly, derivation of rat EG cells were observed solely in the presence of glycogen synthase kinase-3 (GSK3β) and MEK pathway inhibitors. Due to some drawbacks of inhibiting GSK3β molecules such as increases in chromosomal aberrations, in the present study we have attempted to assess a feeder-free protocol that derives EG cells by the simultaneous suppression of TGFβ signaling and the MEK pathway. We have shown that rat EG cells could be generated in the presence of two inhibitors that suppressed the above mentioned pathways. Of note, inhibition of TGFβ instead of GSK3β significantly maintained chromosomal integrity. The resultant EG cells demonstrated the hallmarks of pluripotency in protein expression level and also showed *in vivo* and *in vitro* differentiation capacities.

**Conclusion:**

Rat EG cells with higher karyotype stability establish from PGCs by inhibiting TGFβ and MEK signaling pathways.

## Introduction

Pluripotent stem cells can be helpful tools to answer basic biological questions and study regenerative medicine. The generation of these cells from rats is of great interest due to the physiological and pharmacological similarities of this animal to humans (1).

Although derivation of mouse embryonic stem cells originated three decades ago, until recently the efforts for producing embryonic stem cells from rats were unsuccessful. In 2008 researchers generated rat embryonic stem cells that had the capability of maintaining a pluripotent state if the differentiation pathways, glycogen synthase kinase 3 (GSK3) and mitogen-activated protein kinase (MAPK) were suppressed (2) by selective inhibitors of these pathways, CHIR99021 and PD0325901 (CHIR+PD). It has been well established that mouse primordial germ cells (PGCs) have the ability to dedifferentiate into a pluripotent state - the embryonic germ (EG) cell (3). Earlier efforts for generation of rat EG cells were unsuccessful. However in 2010, CHIR+PD has been shown to induce EG cell generation from E10.5 PGC rat embryos (4). However the exact mechanism of establishing pluripotency under CHIR+PD remains unclear due to a number of drawbacks to GSK3β inhibition. The application of an effective concentration of CHIR leads to partial inhibition of GSK3β whereas higher concentrations solely cause differentiation (2). On the other hand with CHIR+PD, suppression of downstream pathways and substrates is incomplete. Most importantly it has been reported that GSK3β inhibition can trigger chromosomal abnormality due to the controlling effects of this molecule on the dynamic of mitotic spindle microtubules (5). Previously we have shown that the suppression of transforming growth factor-β (TGFβ) and mitogen-activated protein kinase kinase (MEK) signaling pathways using their inhibitors SB431542 (SB) and PD result in efficient production and expansion of mouse ES and mouse EG cells compared to the use of CHIR+PD (6-8). Additionally the resultant cell lines derived under this situation have shown a high percentage of normal chromosomal structure compared to lines derived in the presence of the GSK3β inhibitor. In the present work we attempted to assess the effects of TGFβ and MEK inhibitors on rat EG cell derivation in a defined culture condition. Chromosomal integrity of the resultant EG cells was also analyzed.

## Materials and Methods

### Animals and embryos

In this experimental study, Wistar rat strain (Pasteur Institute, Tehran, Iran) was used for PGC derivation. All embryos were obtained by natural mating. The day that the copulation plug was detected was considered as day 0.5 of gestation. Mouse embryonic fibroblasts (MEF) were obtained from 12.5 day post-coitus (dpc) embryos of the NMRI strain and used as feeder cells. All animal studies received approval from the Royan Institutional Review Board and Ethics Committee.

### Primordial germ cell isolation and embryonic germ cell establishment

PGCs were isolated from 10.5 dpc rat embryos in which all of the extra embryonic parts were removed. The posterior segment at the base of the allantois was detached and digested with trypsin/EDTA (Invitrogen, USA) for single cell production. We cultured the resultant single cells from each embryo on 2 cm^2^ gelatin (Sigma-Aldrich, USA)-coated dishes in day 0 medium which contained knockout Dulbecco’s modifi ed Eagle’s medium (KO-DMEM, Invitrogen), 2% fetal calf serum (FCS, HyClone, USA), 0.1% non-essential amino acids (Invitrogen), 2 mM L-glutamine (Invitrogen), 0.1 mM β-mercaptoethanol (Sigma-Aldrich), 100 U/ml penicillin and 100 mg/ml streptomycin (Invitrogen) supplemented with recombinant mouse leukemia inhibitory factor (LIF, 1000 U/ml, ESGRO, Chemicon), laboratory generated basic fibroblast growth factor (bFGF, 25 ng/ml, Royan Institute, Iran) and stem cell factor (SCF, 100 ng/ml, R&D, USA). After two days, we replaced the medium with a serum-free medium that contained DMEM/F12 (Invitrogen) and neurobasal (Invitrogen) at a 1:1 ratio, 1% N2 supplement (Invitrogen), 1% B27 supplement (Invitrogen), 2 mM L-glutamine, 1% MEM nonessential amino acids, 100 U/ml penicillin, 100 mg/ml streptomycin, 0.1 mM β-mercaptoethanol and 5 mg/mL bovine serum albumin (BSA, Sigma-Aldrich), 1000 U/ml LIF, and small molecules (see below). The medium was renewed every other day. We detected EG cell colonies 7-10 days after beginning the culture. To produce EG cells, the EG colonies were picked and transferred to MEF-covered dishes in the exact medium from which they were derived. After 2 days the entire culture was trypsinized and expanded into EG cells.

### Small molecules

In this study, we used the following small molecules: dimethyl sulphoxide (DMSO, Sigma-Al-drich) as the solvent, PD0325901 (PD, 1 μM, MEK inhibitor, Stemgent, USA), CHIR99021 (CHIR, 3 μM, GSK3 inhibitor, Stemgent) and SB431542 (SB, 10 μM, TGFβ inhibitor, Sigma-Aldrich).

### Antibodies

For immunofluorescence staining EG cells were fixed in 4% paraformaldehyde prepared in phosphate buffered saline (PBS) at pH=7.4 (Invitrogen) for 20 minutes. Then cells were washed twice with 0.1% tween-20 (Sigma-Aldrich) in PBS and permeabilized with 0.2% Triton X-100 (Sigma-Aldrich) in PBS for 20 minutes. To avoid non-specific antibody binding, cells were blocked with 10% goat serum (Sigma-Aldrich) in PBS for 60 minutes. Afterwards, EG cells were incubated overnight in primary antibody solution at 4˚C. The following primary antibodies were used in this study: Oct-4 (1:100, Santa Cruz Biotechnology, SC-5279, USA) and Sox2 (1:100, Santa Cruz Biotechnology, SC-365823) for detection of the pluripotency state. α-Mhc (1:200, Abcam, ab15, USA), Nkx2.5 (1:200, Abcam, ab106923), Tuj1 (1:200, Sigma- Aldrich, T-8660), Map2 (1:200, Sigma-Aldrich, M1406) and GFAP (1:200, Sigma-Aldrich, G3893) were used to detect differentiated cardiac and neural cells. Cells were subsequently washed twice with 0.1% tween-20 in PBS for 10 minutes and incubated with the secondary antibody for 1 hour at 37˚C. Fluorescence-conjugated secondary antibodies used in this study were Alexa fluor 488 goat anti-mouse (1:500, Invitrogen, A-11001) and Alexa fluor 594 goat anti-rabbit (1:500, Invitrogen, A-11037). After washing with PBS that contained 0.1% tween-20 for 5 minutes, the cell nuclei were stained with 4΄,6-diamidino- 2-phenylindole (DAPI, Sigma-Aldrich). The labeled cells were analyzed with a fluorescent microscope (Olympus, Japan). Immunofluorescence staining without primary antibodies was used as the negative control.

### Karyotype assessment

We performed standard G banding to assess chromosome integrity of the EG cells. Initially, synchronization of the EG cell cycle was performed by treatment with 0.66 μM thymidine (Sigma-Aldrich, T1895) at 37˚C. After 16 hours, the medium was replaced by EG cell medium and the cells were allowed to incubate for 5 hours at 37˚C in order to enter metaphase. Subsequently, the cells were arrested in M phase by the application of 0.15 μg/ml colcemid (Invitrogen, 15212-012) for 45 minutes at 37˚C. Then the harvested cells were treated with a hypotonic solution (0.075 M KCl, Merck, Germany) for 10 minutes at 37˚C after which cells were fixed in a 3:1 ratio of ice-cold methanol (Merck, 1.06008) and acetic acid (Merck, 1.00063). Finally, the fixed cells were dropped onto chilled slides and the G banding method was used for chromosomal analysis.

### Directed in vitro differentiation

To assess the direct differentiation toward cardiomyocyte cells, embryonic bodies (EBs) were formed in hanging drops by culturing 800 EG cells in 20 μl of a medium that contained KO-DMEM, 15% FCS, 0.1% MEM non-essential amino acids, 2 mM L-glutamine, 0.1 mM β-mercaptoethanol, 100 U/ml penicillin and 100 mg/ml streptomycin supplemented with 10-4 M ascorbic acid (vitamin C, Sigma-Aldrich, A4403) without LIF (9). The EBs were subsequently cultured for over five days in a suspension mode. To allow for expansion into beating cardiomyocytes, the cells were plated on 1% gelatin-coated tissue culture dishes for an additional seven days.

For differentiation of EG cells into a neural mode, we produced EBs by culturing 200 EG cells in 20 μl of the cardiac differentiation medium. After four days, the medium was refreshed with induction medium that contained DMEM/F12 supplemented with 1% knockour serum replacement (KOSR, Invitrogen), 0.1% MEM non-essential amino acids, 4 mM L-glutamine, 0.1 mM β-mercaptoethanol and retinoic acid (2 μM, Sigma-Aldrich, R2625) for an additional four to six days. Afterwards EBs were plated onto poly- L-lysine-coated dishes in the presence of induction medium and allowed to undergo differentiation into mature neurons for two additional weeks (10).

### In vivo differentiation via teratoma formation

Approximately 2-3×10^6^ EG cells were subcutaneously transplanted into nude mice. We observed teratoma formation at two weeks after injection of the EG cells. The teratomas were isolated and fixed in 4% Bouin’s solution for 48 hour at room temperature. Subsequently the paraffin embedded tissue was sectioned into 6 μm-thick sections which were stained with hematoxylin-eosin. Derivatives of the three germ layers were determined by observation with a bright field microscope.

### Statistical analysis

The data for the efficiency significance of EG cell derivation in different groups were analyzed by generalized linear model (GLM) and the chi square test. P values<0.05 were considered signiﬁcant.

## Results

### Generation of rat embryonic germ cell colonies via SB+PD

**Fig.1:**
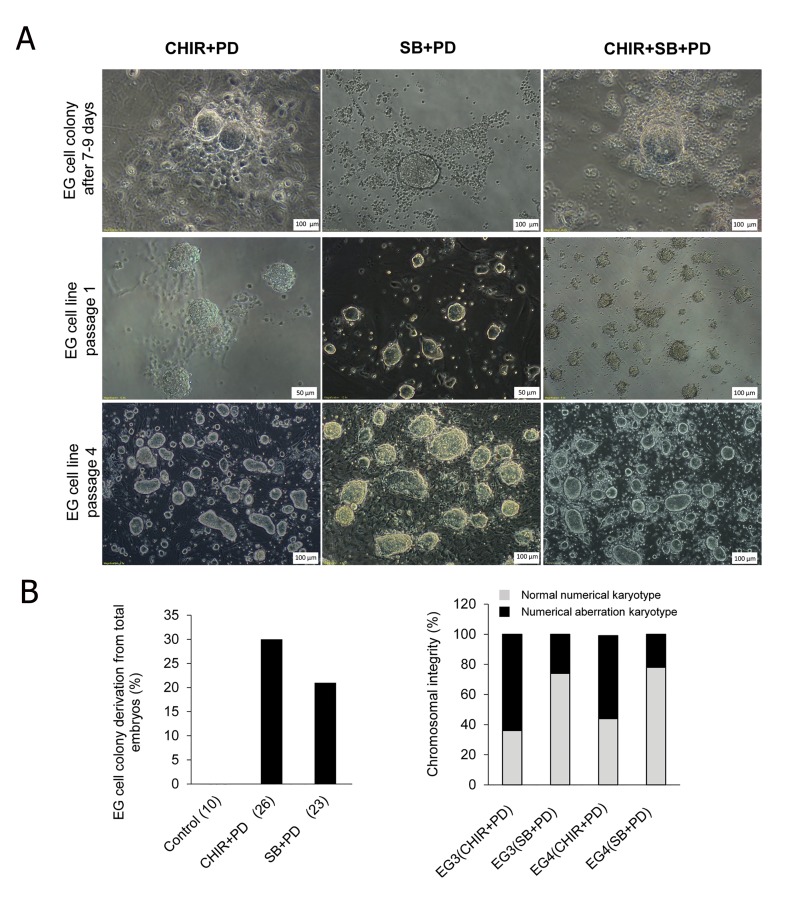
Induction of pluripotency in 10.5 dpc primordial germ cells (PGC) by SB+PD. A. Phase contrast picture of: embryonic germ (EG) cell colonies after 7-10 days, passage 1 and passage 2 EG cell derived in CHIR+PD, SB+PD and CHIR+SB+PD conditions from 10.5 dpc rat embryos, B. The efficiency of EG cell colony derivation from 10.5 dpc rat embryos. The percentage of EG cell colony derivation was calculated relative to the total embryos. The numbers in parentheses indicate the number of embryos tested in that group. All groups contained LIF. The negative control group contained N2B27+LIF+DMSO and C. Karyotype status of two rat EG cells (EG3 and EG4) after 4 passages in CHIR+PD and SB+PD cultures.

After we removed all of the extra-embryonic membranes of the E10.5 embryo, the folded embryo with the allantois that protruded from the posterior area was visualized. The portion at the base of the allantois which contains PGCs was detached from the whole body. After trypsinization, the resultant single cells were cultured on gelatin-coated culture dishes for two days in a medium that contained 2% FCS, SCF and bFGF (day 0 medium). After 48 hours the medium was replaced with a serum-, SCF- and bFGF-free medium that contained LIF plus CHIR+PD or SB+PD for the remaining eight days (Fig.1A). Medium refreshment was performed every other day. After 7-10 days from beginning the culture, we observed round dome-shaped EG colonies in both CHIR+PD and SB+PD media (Fig.1A). In the negative control group which contained no small molecules, there was no EG colony formation after 10 days. The efficiency of colony formation in the CHIR+PD group was 30%, whereas in the SB+PD group was 21% (Fig.1B)

### Generation of embryonic germ cells applying SB+PD

Following the appearance of EG colonies on days 7-10, we hand-picked the colonies and transferred them onto an MEF-coated culture dish with the medium they were derived in. The colonies were maintained in this condition for two days after which the entire culture dish was treated with accutase enzyme to obtain a single cell suspension. The resultant cell suspension then transferred to a new MEF covered dish. After 4-6 days we observed multiple EG colonies in the culture which confirmed the presence of the EG cells at passage one (Fig.1A).

### Passagability of the SB+PD-derived embryonic germ cells

We assessed the passagability of the EG cells in the CHIR+PD and SB+PD groups. Our results showed that EG cells derived under CHIR+PD conditions were capable of maintaining their proliferation up to 10 passages. The SB+PD-derived EG cells maintained a good shape and undifferentiated morphology until the fourth passage (Fig.1A) after which we observed signs of differentiation in these cells. Addition of CHIR to the SB+PD group promoted their passagability up to passage eight. Of note, all of the SB+PD-derived cell lines could be cryopreserved and survived the freeze-thaw process after they were transferred to serum-free SB+PD medium upon thawing.

### Assessment of chromosomal instability

One of the most important criteria for a legitimate cell line is a normal chromosomal content. In this study, we have conducted a parallel experiment to assess the chromosome numbers of two SB+PD-derived EG cells after four passages under SB+PD and CHIR+PD conditions. Our results showed that only 37 and 44% of EG cells cultured in CHIR+PD retained the euploid chromosome complements with 42 chromosomes. However, 74 and 78% of cells cultured in SB+PD showed normal chromosomal content (Fig.1C).

### Characterization of SB+PD-derived embryonic germ cells

**Fig.2:**
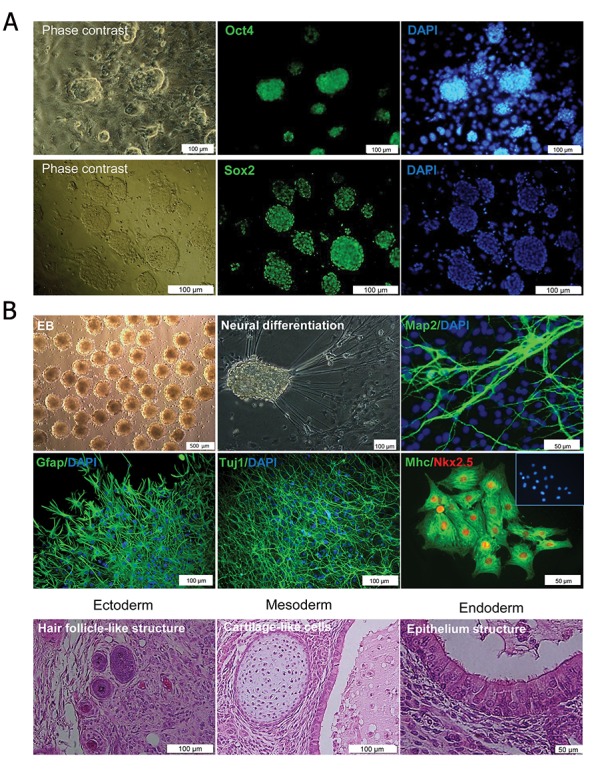
Characteristics of the established (SB+PD)-derived embryonic germ (EG) cells from rats. A. Immunofluorescence labeling for pluripotency markers Oct4 and Sox2 are shown, counterstained for 4′,6-diamidino-2-phenylindole (DAPI). Data are from cells after four passages, B. Evaluation of *in vitro* differentiation capacity in the SB+PD-derived EG cells. Immunostaining for the derivatives of two EG layers after directed differentiation, Tuj1 and Map2 as neuron markers, GFAP as the astrocyte marker, and α-Mhc and Nkx2.5 as cardiomyocyte markers and C. *In vivo* differentiation capacity of SB+PD-derived EG cells by teratoma formation assay. The derivatives of three germ layers are observed in histological sections by hematoxylin-eosin staining.

The obtained SB+PD-derived EG cells were analyzed for pluripotency hallmarks. The EG cells were immunoreactive for Oct4 and Sox2 nuclear factors at the protein level (Fig.2A). A pluripotent cell line should gain differentiation property upon exposure to inductive medium. First, we induced the production of EB from EG cells in the absence of LIF. We showed that EBs could fully differentiate to neuronal cells a few days after induction. Their true neural identity was verified by immunofl uorescent staining as they expressed GFAP (glial marker), Tuj1 and Map2 (both mature neuron markers). The 4-day EBs was transferred onto gelatin-coated dishes in the presence of ascorbic acid. After 10 days the colonies began beating. On day 14 only the beating colonies were chosen and trypsinized. The resultant single cells were maintained in culture dishes for two days before fixation. The mentioned cells were positive for the nuclear cardiac marker Nkx2.5 and cytoplasmic marker MHC (Fig.2B). It has been proven that pluripotency is accompanied by tumorigenicity. To confirm this feature in our cells we subcutaneously transplanted SB+PD-derived EG cells into nude mice. The teratoma emerged after two weeks however we allowed it to grow and differentiate for an additional two weeks before hematoxylin-eosin staining was performed on the teratoma sections. All three germ layers were detectable in the sections in which the hair follicle structure was a representative for ectoderm, cartilage cells for mesoderm and gut-like epithelium for endoderm (Fig.2C). Therefore our SB+PD-derived EG cells had the capability for differentiation both *in vitro* and *in vivo*.

## Discussion

In the present study, we successfully generated EG cells by using inhibitors of the TGFβ and MEK pathways named as SB+PD. Formerly the derivation of EG cells from rats were just observed after inhibition of the GSK3β and MEK pathways by CHIR+PD (4). In that report, SCF was present for the entire cell culture period due to the presence of SCF-producing feeder cells. In the present work we have designed a new culture condition where no feeder layer is used. Thus the PGCs are not exposed to SCF for the entire culture period and instead they receive soluble SCF for just two days. In contrast to the previous report (4) that showed the emergence of EG colonies 10 days after the first passage (24 days from the onset), we derived EG colonies under feeder-free conditions after 7-10 days from the beginning of the culture, prior to passaging. Of note, in another study, emergence of the EG colonies prior to passaging occurred only in the presence of the feeder layer which was eliminated in our culture protocol (11). Additionally, the application of SB+PD from the third day of the PGC culture showed that in order to derive EG colonies, the inhibition of GSK3β molecule is not necessary. We observed the generation of EG colonies in both CHIR+PD and SB+PD combinations with no significant difference in derivation efficiency. Thus SB+PD could possibly be a suitable condition for the generation of EG colonies at almost the same efficiency as CHIR+PD. The EG cells derived in SB+PD were characterized for pluripotency markers and considering the *in vitro* and *in vivo* differentiation assays, we confirmed that the EG cells generated by SB+PD showed stem cell characteristics. Although SB+PD induced PGC reprograming over a short period time, the self-renewability of EG cell lines were maintained for only four passages after which addition of GSK3β inhibitor was necessary for EG cell maintenance. But in the other hand we observed that the majority of cells cultured in SB+PD (76%) showed normal chromosomal content compared to very low percentage of CHIR+PD treated cells (40%). In support of this it has been previously reported that inhibition of GSK3β could induce chromosomal abnormalities due to the important role of this molecule on the dynamics of the metaphasic spindle (5). It was demonstrated that only half of the produced rat EG cell lines retained a stable chromosome number under CHIR+PD conditions (11). Moreover, SB+PD preserve mouse ES cells with higher genomic integrity following long-term cultivation compared with CHIR+PD (8). Our current results showed that although CHIR+PD provided a suitable culture for cell proliferation, the observed numerical abnormality in chromosomes were greater than two times more in cells expanded under this condition compared with cells expanded under SB+PD.

## Conclusion

To our knowledge this is the first report on the derivation of rat EG cells under fully defined conditions with no feeder. Of note, we have introduced a new combination of pathway inhibitors for the reprogramming of rat PGCs which could induce rather efficient EG colony formation. The mentioned combination was supportive of EG cell proliferation up to four passages and most importantly could preserve the line chromosomal stability compared to the formerly used condition. A better comprehension of how the PGCs dedifferentiate under the inhibition of TGFβ and MEK pathways would be helpful toward understanding the reprogramming process in general.
